# Associations between statins and COPD: a systematic review

**DOI:** 10.1186/1471-2466-9-32

**Published:** 2009-07-12

**Authors:** Claudia C Dobler, Keith K Wong, Guy B Marks

**Affiliations:** 1 Woolcock Institute of Medical Research, Sydney, New South Wales, Australia; 2 Department of Respiratory Medicine, Liverpool Hospital, Sydney, New South Wales, Australia; 3 South Western Sydney Clinical School, University of New South Wales, Sydney, Australia

## Abstract

**Background:**

Statins have anti-inflammatory and immunomodulating properties which could possibly influence inflammatory airways disease. We assessed evidence for disease modifying effects of statin treatment in patients with chronic obstructive pulmonary disease (COPD).

**Methods:**

A systematic review was conducted of studies which reported effects of statin treatment in COPD. Data sources searched included MEDLINE, EMBASE and reference lists.

**Results:**

Eight papers reporting nine original studies met the selection criteria. One was a randomized controlled trial (RCT), one a retrospective nested case-control study, five were retrospective cohort studies of which one was linked with a case-control study, and one was a retrospective population-based analysis. Outcomes associated with treatment with statins included decreased all-cause mortality in three out of four studies (OR/HR 0.48–0.67 in three studies, OR 0.99 in one study), decreased COPD-related mortality (OR 0.19–0.29), reduction in incidence of respiratory-related urgent care (OR 0.74), fewer COPD exacerbations (OR 0.43), fewer intubations for COPD exacerbations (OR 0.1) and attenuated decline in pulmonary function. The RCT reported improvement in exercise capacity and dyspnea after exercise associated with decreased levels of C-reactive protein and Interleukin-6 in statin users, but no improvement of lung function.

**Conclusion:**

There is evidence from observational studies and one RCT that statins may reduce morbidity and/or mortality in COPD patients. Further interventional studies are required to confirm these findings.

## Background

Chronic obstructive pulmonary disease (COPD) is a common disease with a high burden to society on a worldwide scale [1]. Only smoking cessation [2] and long-term oxygen therapy, in patients with resting hypoxemia while awake [3,4], clearly alter prognosis for survival or decline in lung function. The lack of potent treatment options for COPD patients contrasts with the development of new treatments in other high burden chronic diseases like cardiovascular disease. Several drugs, in particular statins, have been shown to improve prognosis after acute coronary events during the last 20 years [5].

Recently statins have emerged as a possible disease modifying agent in COPD. The rationale for this at least partly derives from the fact that the pathogenesis of COPD involves inflammatory processes [6], and persistent systemic inflammation seems to be present even in patients with stable COPD who do not currently smoke [7]. Lee et al. showed that simvastatin ameliorated the structural and functional derangement of rat lungs caused by cigarette smoking, partly by suppressing inflammation and matrix metalloproteinase-9 induction and preventing pulmonary vascular abnormality [8]. Statins possess pleiotropic effects in addition to their conventional lipid-lowering properties including anti-inflammatory, antioxidant, antithrombogenic and vascular function-restoring actions [9-11]. For example they have been shown to have a beneficial effect in sepsis and pneumonia [12,13].

There are reports from observational studies that statins may reduce morbidity and mortality in COPD patients. Whether statins have a beneficial effect in COPD patients by primarily reducing cardiovascular complications or because they exhibit an action directly targeting pulmonary inflammation is, however, a matter of controversy. We conducted a systematic review to find evidence for our hypothesis that statin treatment has a disease modifying effect in patients with COPD and improves a) morbidity and b) mortality.

## Methods

### Identification and selection of papers

We searched the published English-language literature to identify studies that examined the effect of statins in patients with COPD. We searched two electronic databases: MEDLINE through OvidSP and PubMED (1950–31^st ^October 2008) and EMBASE through OvidSP (1980–31^st ^October 2008). The following search terms were used to identify citations relevant to statins:

• statins,

• hydroxymethylglutaryl-CoA reductase inhibitors,

• lipid/cholesterol lowering therapy/drugs/medication, and

• generic names of different types of statins

The following search terms were used to identify citations relevant to COPD:

• [chronic] obstructive pulmonary/respiratory/lung disease,

• COPD,

• airflow obstruction, and

• airway/lung/pulmonary inflammation

For details concerning the search strategy see Additional file 1. Additionally the reference lists of identified publications were searched. The search strategy was discussed and agreed upon by all authors, and advice was sought from a medical librarian experienced in medical informatics. The search was limited to articles on research in humans. All types of studies were included.

On reviewing the selected titles and abstracts we retained publications reporting clinical or laboratory outcome measurement in COPD patients treated with statins. Studies in which outcomes of treatment with statins were examined for a subgroup of subjects with COPD or obstructive pulmonary function were also included. Only articles reporting original data were retained; abstracts, editorials and letters were excluded.

### Review of studies

The initial database created from the electronic searches of MEDLINE and EMBASE was compiled with EndNote and all duplicate citations were eliminated. Two reviewers (CCD and KKW) screened these citations by title and abstract review to identify potentially relevant studies. The full text of these papers were then retrieved and reviewed to confirm eligibility. Disagreements between the reviewers were resolved by consensus. Studies were eligible for inclusion if they were primary articles reporting an association between statin treatment and clinical or laboratory outcomes in COPD patients.

### Data extraction

We extracted information on study objective, study design, inclusion and exclusion criteria for participants, COPD definition for the study purpose, details on statin treatment, use of corticosteroids, smoking status, cardiovascular comorbidities in participants, duration of follow-up, and outcome measurements. Specific attention was given to the inclusion or exclusion of patients with asthma. Details on statin treatment included type of statin, dosage, duration of treatment and adherence to treatment.

### Assessment of methodologic quality of included studies

The RCT was assessed for evidence of concealed randomization, similarity of the randomized groups at baseline, standardization of non-intervention treatment strategies between treatment groups, blinding of patients and investigators, number of crossovers, intention-to-treat analysis, follow-up to the defined outcome, and generalizability of the conclusions of the trial to other populations [14]. Observational studies were evaluated for internal validity based on adequate description of patient characteristics (including age, definition of COPD and cardiovascular comorbidities), adequate description of treatment strategy (statin type, dosage, duration of treatment) and follow-up. They were also assessed for external validity using a qualitative determination of the degree to which the findings of the study could be generalized to other populations [15].

## Results

**Figure 1:**
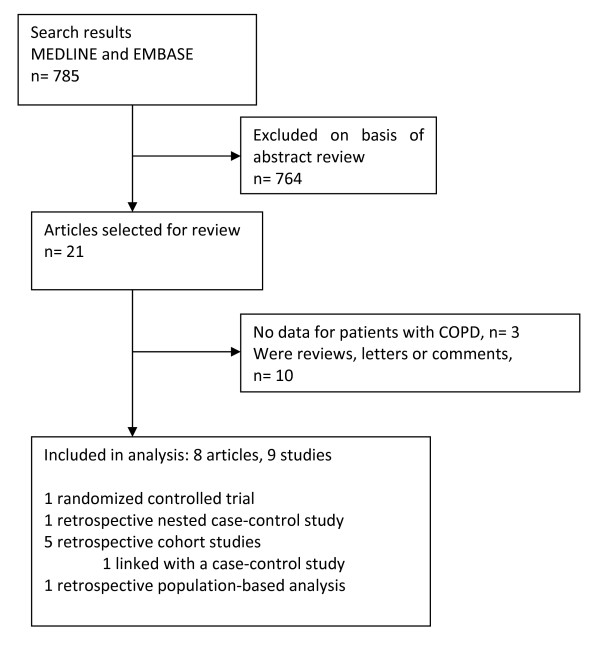
**Study selection process**.

Overall, 785 citations were identified, from which 21 articles were selected for review (Fig 1). Of these, 10 were excluded because they were reviews, letters or comments. Three original articles did not meet the inclusion criteria. One study, which examined the effect of statin use on lung function in elderly patients with various smoking histories, was excluded, because there was no specific analysis for patients with COPD or obstructive pulmonary function test findings published [16]. We excluded one study that examined the effect of statins in patients with asthma [17], and another study that reported the effects of statin use on lung transplant recipients [18]. A total of 8 papers reporting 9 studies met the inclusion criteria (Table 1). They were all published between 2006 and 2008 and reported effects of statin treatment on patients with COPD.

**Table 1:** Characteristics of included studies

**Author, place, date of publication**	**Study design**	**Study purpose**	**Statin exposed group**	**Controls**	**Outcome measurements**
Lee T-M, et al., Taiwan, 2008[19]	Randomized, double-blinded, placebo-controlled trial	To investigate whether pravastatin administration is effective in improving exercise capacity in patients with COPD, and whether baseline or serial changes in hs-CRP over time are associated with corresponding changes in exercise capacity.	n = 62 patients with clinically stable COPD, received pravastatin (40 mg/day) over a period of 6 months (randomly assigned, double blind).	n = 63 patients with clinically stable COPD, received placebo over a period of 6 months (randomly assigned, double blind).	Exercise capacity CRP/IL-6 Secondary outcome measurements: Lung function Borg dyspnea score after exercise tests

Blamoun AI et al, USA, 2008 [27]	Cohort study	To assess the rate of COPD exacerbations and intubations in COPD patients taking statins	n = 90 patients with primary or secondary diagnosis of COPD who were taking statins at the time of hospital admission and during the 1-year follow-up	n = 95 patients with primary or secondary diagnosis of COPD who were not taking statins at the time of hospital admission and during the 1-year follow-up	COPD exacerbations Intubations secondary to COPD exacerbation

Van Gestel YR et al., Netherlands, 2008 [21]	Cohort study	To examine the relation between statins and mortality (within 30 days and 10 years) in a group of patients who underwent surgery for peripheral arterial disease and compare results in those with versus without associated COPD	COPD group: n = 330 COPD patients who underwent elective vascular surgery and who did use statins	COPD group: n = 980 COPD patients who underwent elective vascular surgery and who did use statins	All-cause mortality, short- and long-term (within 30 days and 10 years of follow-up respectively)

Søyseth V et al., Norway, 2007[22]	Cohort study	To determine whether statins alone or in combination with inhaled steroids improve survival after COPD exacerbation	n = 118 patients with a diagnosis of COPD exacerbation at hospital discharge who were taking statins at the time of discharge	n = 736 patients with a diagnosis of COPD exacerbation at hospital discharge who were not taking statins at the time of discharge	All-cause mortality

Frost FJ et al., USA, 2007 [20]	Cohort study, and separate case-control studies (for influenza and COPD deaths)	To assess whether statin users have reduced mortality risks from influenza and COPD	Cohort study: n = 19,058; patients with statin exposure Case control study: n = 207; COPD deaths (in hospital)	Cohort study: n = 57,174; patients with no history of statin therapy Case-control study: n = 9,622; surviving patients with either an inpatient or outpatient diagnosis of COPD	Mortality from COPD (and influenza, not included in this review)

Keddissi JI, et al., USA, 2007 [26]	Cohort study	To assess the ability of statins to preserve lung function in current and former smokers and to reduce the incidence of respiratory-related urgent care	n = 215; statin users who were smokers or ex-smokers and had abnormal baseline spirometry (majority with obstructive spirometry findings, but restrictive findings also included).	n = 203; non-statin users who were smokers or ex-smokers and had abnormal baseline spirometry (obstruction or restriction)	Lung function (annual decline in FEV1 and FVC) Respiratory-related ED-visits and hospitalizations

Mancini GB et al., Canada, 2006 [25]	Nested case-control study (time-matched)	To determine if statins, angiotensin-converting enzyme-inhibitors and angiotensin receptor blockers reduce total mortality, COPD hospitalisations and myocardial infarctions in COPD patients	Two distinct COPD cohorts: 1) n = 2983 (sum of cases analysed for different endpoints, n = 3231 when steroid users included), high cardiovascular risk cohort (COPD patients having undergone coronary revascularization) 2) n = 7617 (sum of cases analysed for different endpoints, n = 8240 when steroid users included), low cardiovascular risk cohort (COPD patients without previous myocardial infarction and newly treated with nonsteroidal anti-inflammatory drug)	from same databases as study population, matched for age and year of cohort entry and still at risk of the event (endpoint) n = 59,170 for cohort 1 (sum of controls for different endpoints, n = 64,185 including steroid users) n = 152,177 for cohort 2 (sum of controls for different endpoints, n = 164,672 including steroid users)	COPD hospitalizations Myocardial infarction All-cause mortality

Ishida W, et al., Japan, 2007 [23]	Ecological analysis	To assess effects of statin use on mortality from major causes of death (cardiovascular diseases, COPD, pneumonia etc.)	COPD deaths in the >65 yrs old population in each of the 47 prefectures of Japan	No control	Mortality from COPD (and other major diseases), related to statin sales in the same area

Only one study was a randomized controlled trial (RCT). The other studies were analyses of observational data and included one nested case-control study, five historical cohort studies of which one was linked with a case-control study, and one ecological study.

### Treatment outcomes

**Figure 2:**
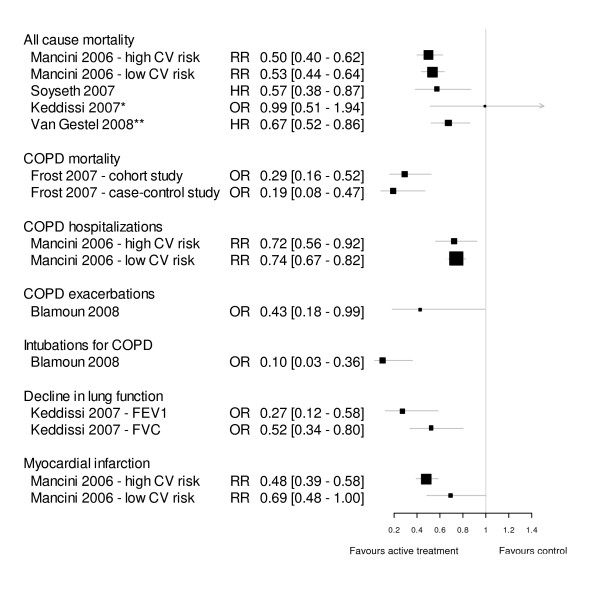
**Forest plot of effect estimates of statins for ORs, HRs and RRs**. RR = Risk ratio, HR = Hazard ratio, OR = Odds Ratio. Where available, adjusted estimates (OR, HR, RR) were used. Values less than 1 indicate a better outcome with statin therapy. Box size is proportional to precision of the estimate. * Mortality was only given for the whole cohort, which included 24% patients with restrictive rather than obstructive spirometry finding. ** 10-year mortality (mortality at 30 days not shown).

Data on the association of statin treatment with continuous outcome variables are presented in Table 2. Effect estimates for binary outcomes are presented in Fig 2. Three cohort studies reported decreased all-cause mortality in patients treated with statins, whereas no difference in all-cause mortality between statin users and non-statin users was found in one cohort study. Another cohort study and the linked case-control study showed a decrease in deaths attributed to COPD. The ecological analysis found less COPD mortality in areas with high statin use. A reduction in incidence of respiratory-related urgent care with statin use was found in two studies. One cohort study found fewer COPD exacerbations and fewer required intubations secondary to COPD in patients who were taking statins. One observational study reported attenuated decline in pulmonary function parameters in statin users, whereas the only interventional study did not find a difference in lung function in statin users after six months of treatment. However, the RCT reported improvement in exercise capacity and dyspnea after exercise associated with decreased levels of CRP and IL-6 in statin users.

**Table 2:** Association of statin treatment with continuous outcome variables

**Study**	**Outcome**	**Parameter estimate and 95% confidence interval**
**Mortality 2° to COPD**		

Ishida W, et al., Japan, 2007 [23]	Decreased mortality 2° to COPD in statin users	Inverse correlation between statin prescriptions dispensed and mortality due COPD by prefecture, p < 0.0001

**Respiratory-related urgent care**		

Keddissi JI, et al., USA, 2007 [26]	Reduction in respiratory-related emergency-department-visits and/or hospitalizations in statin users	Incidence of respiratory related urgent care, obstructive spirometry group 0.12 ± 0.29/patient-yrs in statin users versus 0.19 ± 0.32/patient-yrs in control, p = 0.02

**Lung function**		

Keddissi JI, et al., USA, 2007 [26]	Lower decline in FEV1 and FVC/yr in statin users	Obstructive spirometry group change in FEV1 +5 ± 207 ml/yr in statin users change in FEV1–86 ± 168 ml/yr in control (p < 0.0001) change in FVC +33 ± 452 ml/yr in statin users change in FVC-150 ± 328 ml/yr in control (p < 0.0001)

Lee T-M, et al., Taiwan, 2008 [19]	No difference in pulmonary function parameters in statin users	Pravastatin group: FEV1% at baseline 51 ± 18, at follow-up 55 ± 19 Placebo group: FEV1% at baseline 56 ± 13, at follow-up 55 ± 14, p > 0.05

**Exercise capacity**		

Lee T-M, et al., Taiwan, 2008 [19]	Improvement in exercise time in statin users	Pravastatin group: exercise time in s at baseline 599 ± 323, at follow-up 922 ± 328 Placebo group: exercise time in s at baseline 608 ± 273, at follow-up 609 ± 180, p < 0.05

**Borg dyspnea score after exercise tests**		

Lee T-M, et al., Taiwan, 2008 [19]	Lesser degree of dyspnea after exercise in statin users	Pravastatin group: Borg dyspnea score at baseline 7.0 ± 0.8, at follow-up 4.0 ± 0.7 Placebo group: Borg dyspnea score at baseline 6.9 ± 0.8, at follow-up 6.9 ± 1.0, p < 0.05

**CRP/IL-6 levels**		

Lee T-M, et al., Taiwan, 2008 [19]	Decrease in CRP/IL-6 levels in statin users	Pravastatin group: CRP (mg/l) at baseline 3.94 ± 3.54, at follow-up 3.85 ± 2.56 Placebo group: CRP (mg/l) at baseline 4.06 ± 2.67, at follow-up 2.66 ± 2.49, p < 0.05

Due to a high degree of heterogeneity of study designs and outcome measurements a meta-analysis of the reviewed data was not feasible.

### Assessment of methodologic quality of included studies

The RCT [19] described allocation concealment (sealed envelopes) and similarity of the groups at baseline. Treatment strategies were standardized between the treatment groups, patients and investigators were blinded and an intention-to-treat analysis was performed. Subject retention to outcome assessment was 86% after 6 months (18 out of 125 patients withdrew during the study).

The observational studies that were analysed all had adequate description of patient characteristics including details on patients' age and definition of COPD for study purposes. All studies (except for the study by Frost et al. [20]) described cardiovascular comorbidities. Descriptions of treatment strategies lacked details regarding type of statin used and dosage. The study by van Gestel et al. [21] had the most comprehensive description of treatment strategy and also documented very good follow-up of 96%. The issue of external validity or generalizability of the findings is dealt with in the next section.

### Key differences between included studies

#### Definition of COPD

Different criteria were applied for definition of COPD. The majority of studies relied on a patient chart diagnosis of COPD. Those studies using health system data identified COPD based on one or more of the following recorded codes from the International Classification of Disease: ICD-10 J41–44 or ICD-9 490–496 [20,22,23]. In one of these studies spirometric data were also available and only 88% had a FEV1/FVC ratio < 0.7 [22], which means that 12% of the cohort patients with spirometry results available did not meet the GOLD criteria [24] for the definition of COPD. Patients with COPD where identified through medication use in one study [25]. The cohort study by Keddisssi et al. [26], the study by van Gestel et al. [21] and the RCT [19] applied lung function criteria (FEV1/FVC<70%) for the definition of obstructive lung disease. However, in the study by Keddissi et al. there was a discrepancy between 169 patients (40%) that had a COPD diagnosis based on information from medical records, compared to 319 patients (76%) who were found to have obstructive baseline spirometry. Hence, "COPD populations" analysed in those studies that did not use lung function criteria may differ from people with COPD defined in accordance with GOLD criteria [24].

#### Exclusion of asthma patients

Three studies used specific exclusion criteria to avoid inclusion of subjects who had a primary diagnosis of asthma. Mancini et al. [25] excluded all patients who had used at least one prescription of an inhaled steroid, nasal steroid, and other drugs including nedocromil, ketotifen, and cromoglycate during the year preceding cohort entry. However, they performed a separate analysis for a COPD cohort including users of oral or inhaled steroids (but not nasal steroids). Results were similar when steroid users were included. In the study by Keddissi et al. [26] patients with a clinical history of asthma were excluded. Yet, as the authors of the paper state, it was still possible that some included patients had an asthmatic component to their lung disease, particularly when taking into account that 3–6% of all cohort patients were receiving leukotriene inhibitors. Lee et al. [19] excluded patients with one or more of the following features (possibly indicating a diagnosis of asthma): a history of perennial allergic rhinitis, periodic wheezing and an improvement in FEV1 of >15% after inhalation of a bronchodilator.

#### Concomitant use of steroids

A number of studies examined whether the inclusion of corticosteroid-steroid users altered the findings. Other simply reported on the use of corticosteroids in the study population. The studies by Mancini et al. [25]and Søyseth et al. [22] showed that use of inhaled corticosteroids did not modify the effect of statins on mortality (or the other outcomes measured in Mancini's study). However, the study by Søyseth et al. reported an additive benefit on mortality when statins and inhaled corticosteroids were combined. In the lung function study by Keddissi et al. [26] there was no difference in the use of inhaled or systemic corticosteroids or immunosuppressive therapy between the statin group and the controls. For the whole cohort (including 76% with obstructive pulmonary function test findings and 24% with restrictive pulmonary function test patterns) the change in FEV1 was associated with both statin use and the use of steroids. The change in FVC was associated with statin use, the use of nonsteroidal anti-inflammatory drugs/aspirin and the use of β-blockers. In the RCT from Taiwan [19] 48% of patients in the pravastatin group and 52% in the placebo group were described as steroid dependent at baseline. All medications for COPD were kept constant throughout the study period of 6 months. Modification of the effect of statins by corticosteroids was not evaluated.

#### Smoking status

The smoking status of participants was reported in 5 studies. The study by Søyseth et al. [22] included 6.9% of patients who had never smoked, 37.5% former smokers, 51.8% current smoker and 3.9% had missing data. No definition for who qualified as former smoker (duration of smoking abstinence) was given. The age-adjusted relative mortality was lower in statin users than nonusers in the subgroups of never smokers and current smokers, but not former smokers. Keddissi et al. [26] excluded never smokers in their study. Sixty-five % of their study participants were ex-smokers (defined as patients that quit smoking at least 6 months prior to the last pulmonary function test), 35% were current smokers. The beneficial effect of statins on lung function decline was apparent in both the current smoker and the ex-smoker groups with no significant difference between the groups. The cohort study that analysed exacerbations and intubations for exacerbations of COPD [27], the study that looked at mortality in COPD patients that underwent elective vascular surgery [21] and the interventional study by Lee et al. [19] gave information on smoking status of participants, but did not include subgroup analysis based on smoking status.

#### Cardiovascular risk profile

In the RCT by Lee et al. [19] no characteristics of the patients regarding cardiovascular risks were described. Mancini et al. [25] looked at COPD patients with different cardiovascular risk profiles. One cohort consisted of revascularized patients (percutaneous coronary angioplasty and/or bypass grafting), whereas a second cohort specifically excluded any patients with a myocardial infarction in the five years preceding cohort entry. The study found that the risk reduction for COPD hospitalizations and all-cause mortality with statin treatment was similar in both groups. However, there was no apparent beneficial effect on prevention of myocardial infarction in the low cardiovascular risk group. In the Norwegian retrospective cohort study by Søyseth et al. [22] nearly 30% of all study patients had diagnosed ischaemic heart disease with a significantly higher proportion in the statin group (approximately 60%). About 20% of all patients had congestive heart failure. Adjusted mortality was lower in statin users than non-users in both subgroups and most of the other comorbidity subgroups. No effect modification on statins by ischaemic heart disease or congestive heart failure was shown.

#### Statin treatment

Information on statin treatment including type of statin, dosage, treatment duration and adherence to treatment was very variable.

Three of the observational studies gave details on the types of statins used. In the study by Keddissi et al. [26] approximately 80% of statin users received simvastatin, and the remainder received lovastatin, atorvastatin and fluvastatin. In the study by Blamoun et al. [27] atorvastatin was the most common statin used (52%), followed by simvastatin (24%), lovastatin (10%), pravastatin (8%) and fluvastatin (6%). A third cohort study stated that statins included fluvastatine, simvastatin, pravastatin, atorvastatin, and rosuvastatin with no information on the distribution [21]. The Japanese population-based analysis stated that during the study period pravastatin, simvastatin, atorvastatin, and fluvastatin were commercially available in Japan, but no information on relatively prevalence of use was provided [23].

The study by Frost et al. which included all drugs in the statin class was one of two retrospective studies to include information about the dose of statins used [20]. The usual minimum prescribed dose was found to be 10 mg/d. Statin exposure to any statin was classified into low daily dose (<4 mg/d) and moderate daily dose (≥ 4 mg/d) for any type of statin, averaged over a three month to one year period. The fact that the cut off between low and moderate daily dose lay below the minimum prescribed dose indicated poor compliance. This study suggested a dose-dependent variation of response. The Dutch cohort study used a more sophisticated dosage classification [21]. The dosage of statin therapy was converted to no dose, low dose (<25%), and intensified dose (≥ 25%) of the maximum recommended therapeutic dose. Low dose statin treatment had no beneficial effect on short-term mortality in COPD patients. However, an intensified dose was associated with improved short-term survival. Both low-dose and intensive statin therapy were associated with improved long-term survival in patients with COPD. In the RCT the treatment group received pravastatin 40 mg daily over 6 months [19]. In the restrospective studies duration of treatment varied from at least one prescription in the 60 days prior to the index date [25] to at least 90 days of cumulative statin exposure prior to death or withdrawal [20]. Any FDA approved statins had to be used for at least 3 months prior the last pulmonary function test in the study by Keddissi et al. [26]. This study described a trend towards an association between the change in FEV1 and FEV and the duration of treatment with statins, but this did not reach statistical significance.

## Discussion

The aim of this review was to evaluate evidence of beneficial effects of statin treatment in patients with COPD to determine implications for future studies. We assessed current evidence that statins may alter the natural course of COPD. We identified several observational studies that suggest a benefit of statin treatment on various clinically-relevant endpoints including all-cause mortality, deaths from COPD, respiratory-related urgent care, COPD exacerbations, intubations for exacerbations of COPD and lung function decline in COPD patients. There is evidence from one randomized controlled trial that exercise capacity is increased and dyspnea after exercise is decreased in COPD patients treated with statins.

There are certain limitations with the present systematic review. We deliberately did not use stringent selection criteria as we were aware that current evidence from research in this area is sparse. This resulted in heterogeneity across the selected studies with respect to study design, target population (different definitions for COPD), interventions (statin types, dosage, duration of treatment) and outcomes assessed. We did not include non-English-language papers, thereby possibly limiting the scope of included studies. As in any systematic review, publication bias was a concern, possibly leading to overestimation of the associations of statin treatment with favourable outcomes in COPD.

One important question, which is a matter of ongoing debate, is whether statins exhibit a beneficial effect in COPD because they affect cardiovascular comorbidities as opposed to having a direct disease-modifying effect on COPD. Søyseth et al. hypothesized that statins might improve all-cause mortality in COPD because many COPD patients probably have unrecognized ischaemic heart disease [22]. Cardiovascular disease is increased in COPD patients due to various shared risk factors (e.g., smoking, obesity, diabetes), and there also seems to be a possible synergy between cardiovascular events and pulmonary inflammation [28]. The inflammation that is associated with atherosclerosis and atherothrombosis may be worsened by the systemic inflammatory component of COPD. Studies that have examined the effects of air pollution have previously suggested an association between airways inflammation and cardiovascular events [29,30]. However, the finding by Keddissi et al. that use of statins was associated with an attenuated decline in lung function and a lower incidence of respiratory-related ED-visits and/or hospitalizations in patients with obstructive lung disease implies that statins may have a direct disease-modifying effect on COPD [26].

It is assumed that the observed benefits of statins in patients with COPD derive at least partially from the drugs' anti-inflammatory properties. However, none of the analysed retrospective studies that assessed the effect of statins on outcomes in COPD has examined the correlation between change in inflammatory markers and those outcomes. In their RCT Lee et al. found that pravastatin caused a significant mean decrease in hs-CRP over the course of the study, but about a fifth of patients actually had an increase in hs-CRP. As expected, they found evidence of a floor effect. That is, those with higher baseline CRP levels (>3 mg/L) had a significant decrease in hs-CRP after statin treatment, whereas no changes in hs-CRP was observed in patients with low baseline CRP levels (<3 mg/L)[19]. In this study, significant improvement in exercise capacity in the statin treatment group correlated with decreasing CRP levels. However, no effect of pravastatin on lung function decline was found. The lack of effect on lung function in this RCT contrasts with observed inverse relationship between CRP and lung function in cross-sectional studies[31-33]. It is possible that the study period of 6 months was too short to reveal a significant effect of statins on lung function in this trial.

There are data to show that CRP levels are a predictor of COPD morbidity and mortality [34], and statins have been shown to reduce serum levels of CRP [35]. COPD patients with high baseline CRP levels (in a stable condition) could therefore be a subgroup to benefit most from statin treatment not just in regard to attenuated lung function decline, but also improved mortality. The recently published JUPITER study showed that rosuvastatin significantly reduced the incidence of major cardiovascular events in apparently healthy persons without hyperlipidemia but with elevated CRP levels [36]. However, besides the anti-inflammatory action, other possible effects of statins, e.g. anti-oxidant action, may be partially responsible for the apparent beneficial effect in COPD patients.

Current evidence is insufficient to determine whether smoking status influences the beneficial effects of statin therapy. Yet interestingly, present data, including the study by Alexeeff et al. [16], suggest that statins exhibit beneficial effects in current smokers as well as those who are not currently smoking.

Different statins could possess different modes of action, with resulting variations in outcomes. The lack of information on the effects of specific statins in most of the reviewed observational studies precludes further detailed analysis. Kiener et al. showed that the differential actions of statins are, in part, related to their lipophilicity. Lipophilic statins such as simvastatin and atorvastatin have the greatest anti-inflammatory potential [37]. No difference in FVC or FEV1 decline was seen between the different statins in the study by Keddissi et al. where 80% of patients received simvastatin, and the other patients received either lovastatin, atorvastatin or fluvastatin [26]. However, all those statins are lipophilic, whereas pravastatin, which was used in the RCT by Lee et al. [19] is hydrophilic. This might be a reason for the different effects of statin treatment on lung function decline in those two studies.

The relation of duration and dose of statin therapy to clinical outcomes in COPD or anti-inflammatory effects is unclear. A study of 107 hypercholesterolemic patients treated with simvastatin for 6 weeks showed a significant decline in cytokine levels; however, greater reductions were observed after 6 months [38]. Keddissi et al. described a trend towards an association between the change in FEV1 and FEV and the duration of treatment with statins, but this did not reach statistical significance. Although one of the studies demonstrated a dose-dependent gradient in response [20], the precise dose dependency of effect remains unclear. Also, because statin therapy compliance is thought to be poor [39], assessment of adherence to treatment (e.g. by measuring lipid profiles) is essential in statin studies.

## Conclusion

In summary, our review shows that treatment with statins may have beneficial effects in patients with COPD, possibly reducing all-cause mortality, deaths from COPD, respiratory-related urgent care, COPD exacerbations, intubations for exacerbations of COPD and lung function decline and improving exercise capacity. While statins seem to influence systemic inflammation and cardiovascular morbidity in COPD patients, it appears likely that they also directly target airways inflammation. Types of statins, dosage and treatment duration necessary to exhibit a pleiotropic effect remain unclear. Most of the available data are based on observational studies and randomized controlled trials are urgently needed to evaluate the therapeutic effect of statins in COPD.

## Abbreviations

COPD: chronic obstructive pulmonary disease; CRP: C-reactive protein; ED: Emergency Department; FEV1: forced expiratory volume in 1 second; FVC: forced vital capacity; HR: Hazard ratio; hs-CRP: high-sensitivity C-reactive protein; OR Odds ratio; RCT Randomized controlled trial; RR: Risk Ratio.

## Competing interests

The authors declare that they have no competing interests.

## Authors' contributions

CCD and GBM contributed to the design of the study, data analyses and write-up of the manuscript. KKW contributed to data analyses and write-up of the manuscript. CCD and KKW collected the data. All authors read and approved the final manuscript.

## Pre-publication history

The pre-publication history for this paper can be accessed here:



## Supplementary Material

Additional file 1 **Literature search strategy**. The data provided represent the Ovid SP literature search strategy for Medline and Embase. Click here for file
