# Supplementary material for: Associations between statins and COPD: a systematic review
Source: BMC Pulm Med. 2009 Jul 12;9:32. doi: 10.1186/1471-2466-9-32 (PMC2716302; doi:10.1186/1471-2466-9-32)
Supplement: Additional file 1 — Literature search strategy. The data provided represent the Ovid SP literature search strategy for Medline and Embase. [file 1471-2466-9-32-S1.doc]

**Literature search strategy**

1. hydroxymethylglutaryl-coa reductase inhibitors/ or lovastatin/ or meglutol/ or pravastatin/ or simvastatin/
2. atorvastatin.mp.
3. simvastatin.mp.
4. pravastatin.mp.
5. lovastatin.mp.
6. fluvastatin.mp.
7. statin$.mp
8. (cholesterol lowering adj3 drug*).mp.
9. (cholesterol lowering adj3 medication*).mp.
10. (cholesterol lowering adj3 therap*).mp.
11. (lipid lowering adj3 drug*).mp.
12. (lipid lowering adj3 medication*).mp.
13. (lipid lowering adj3 therap*).mp.
14. 1 or 2 or 3 or 4 or 5 or 6 or 7 or 8 or 9 or 10 or 11 or 12 or 13
15. lung diseases, obstructive/ or asthma/ or bronchitis/ or bronchiolitis/ or bronchitis, chronic/ or pulmonary disease, chronic obstructive/ or pulmonary emphysema/
16. OBSTRUCTIVE LUNG DISEASE.mp. or Lung Diseases, Obstructive/
17. (chronic obstructive adj3 airway* disease*).mp.
18. (chronic obstructive adj3 lung* disease*).mp.
19. (chronic obstructive adj3 pulmonary* disease*).mp.
20. COPD.mp.
21. COAD.mp.
22. airflow obstruction*.mp.
23. (obstructive adj3 airway* disease*).mp.
24. (obstructive adj3 lung* disease*).mp.
25. (obstructive adj3 pulmonary* disease*).mp.
26. (lung* adj3 inflammat*).mp.
27. (airway* adj3 inflammat*).mp.
28. (pulmonary adj3 inflammat*).mp.
29. asthma*.mp.
30. pulmonary function*.mp.
31. respiratory function*.mp.
32. lung function*.mp.
33. 15 or 16 or 17 or 18 or 19 or 20 or 22 or 23 or 24 or 25 or 26 or 27 or 28 or 29 or 30 or 31 or 32
34. 14 and 33
35. limit 34 to human
